# Knowledge, attitudes and willingness to organ donation among the general public: a cross-sectional survey in China

**DOI:** 10.1186/s12889-022-13173-1

**Published:** 2022-05-09

**Authors:** Xiaojing Fan, Meng Li, Heike Rolker, Yingying Li, Jiaoyang Du, Duolao Wang, Enchang Li

**Affiliations:** 1 School of Public Policy and Administration, Xi’an Jiaotong University, No. 28 Xianning West Road, Xi’an, 710049 Shaanxi China; 2 School of Marxism, Zhejiang Chinese Medical University, No. 548 Binwen Road, Binjiang District, Hangzhou, 310053 Zhejiang China; 3 Faculty of Epidemiology and Population Health, London School of Hygiene and Tropical Medicine, London, WC1E 7HT UK; 4 Centre of Health and Bioethics Research, Wenzhou Medical University, Wenzhou Chashan Higher Education Park, Wenzhou, 325035 Zhegjiang China; 5 Department of Epidemiology and Health Statistics, School of Public Health, Xi’an Jiaotong University Health Science Center, No. 76 Yanta West Road,, Xi’an, Shaanxi 710061 PR China; 6 Department of Clinical Sciences, Liverpool School of Tropical Medicine, Liverpool, L3 5QA UK

**Keywords:** Organ donation, Knowledge, Attitudes, Willingness, China

## Abstract

**Background:**

The purpose of this study is to assess the level of knowledge, attitudes, and willingness to organ donation among the general public in China.

**Methods:**

The study population consisted of 4274 participants from Eastern, Central and Western China. The participants’ knowledge, attitudes and willingness to organ donation were collected by a self-designed questionnaire consisting of 30 items. Knowledge is measured by 10 items and presented as a 10 point score, attitudes is measured by 20 items using a 5-step Likert scale and total score ranged between 0 and 80; while the willingness to donate is assessed as binary variable (0 = No; 1 = Yes). A logistic regression model was used to assess the association of knowledge and attitudes with willingness to organ donation, controlling for demographic and socioeconomic confounders.

**Results:**

The questionnaire response rate was 94.98%. The mean score (± SD) of the general public’s knowledge to organ donation was 6.84 ± 1.76, and the mean score (± SD) of attitudes to organ donation was 47.01 ± 9.07. The general public’s knowledge and attitudes were the highest in Eastern China, followed by West and Central China. The logistic regression model indicated a positive association between knowledge and the willingness to organ donation (OR = 1.12, 95%CI: 1.08, 1.17; *P* < 0.001); attitudes were also positively potential determinant of more willingness to organ donation (OR = 1.08, 95%CI: 1.07, 1.09; *P* < 0.001).

**Conclusions:**

Knowledge and attitudes were found to be positively associated with the Chinese general public’s willingness to organ donation. Knowledge about the concept of brain death and the transplant procedure may help raise the rate of willingness to organ donation.

**Supplementary Information:**

The online version contains supplementary material available at 10.1186/s12889-022-13173-1.

## Background

For patients with end-stage organ failure, an organ transplantation is a well-established and often the only life-saving treatment [1]. Globally, the number of patients on a waiting list to receive an organ transplantation exceeds the number of organs donors [2, 3]. The WHO Global Observatory on Donation and Transplantation recently estimated that over 130,000 solid organ transplantations were performed across the globe in 2017, which represents less than 10% of the global need [4]. The provision of deceased donor transplants was shown to be positively associated with per capita gross national income [5, 6]. According to the International Registry in Organ Donation and Transplantation (IRODaT), the countries with the highest number of deceased organ donations in 2019 were Spain, the USA, France, the United Kingdom and Australia, with 48.9 per million population (pmp), 36.88 pmp, 33.25pmp, 24.88 pmp and 22.17 pmp, respectively. The countries with the lowest numbers of organ transplantations in contrast were China (4.43 pmp), Thailand (3.66 pmp), Japan (0.75 pmp), and India (0.65 pmp) [7].

Knowledge and attitudes towards organ donations are determinants of the willingness to donate an organ. In an online survey of 1945 Intensive Care Unit (ICU) nurses, health science students and non-health science students in Austria, Stadlbauer V et al. showed that the study participants knowledge of Austrian organ donation legislation was high [8]. In a cross-sectional study survey of 1275 hospital medical and non-medical staff in 15 Japanese medical facilities, Murakami M et al. found high knowledge about organ donation and transplantation was associated with willingness to become an organ donor [9]. Further, in a survey of 724 physicians of different specialties in the USA, Alkhatib AA et al. showed physicians who were identified as donors were more aware about issues related to organ shortage [10].

In China, organ transplantations have been conducted since the 1960s and have saved tens of thousands of patients. Today, China is the country with the second highest number of organ transplantations worldwide and completed 6,302 organ donations in 2018 [11, 12]. Yet, there is still a shortage of organ donors [13]. A survey among 373 health professionals from 7 hospitals in Dalian and 1 hospital in Chaozhou in China, showed that health professionals lacked knowledge about organ donation on the sector where to donate organ and the procedure of donation [14]. Attitudes of visitors at adult intensive care unit to organ donation is low in Hong Kong [15]. To date, research is limited to studies with small sample size and lacks geographic diversity.

The aim of this study is to assess the knowledge, attitudes, and willingness to organ donation among the general public in China. An analysis of the knowledge and attitudes towards organ donation in the general Chinese public is required to improve the knowledge about organ donation as well as inform policy and legislation aimed at increasing the number of organ donation.

## Methods

### Study population

We conducted a survey among residents in 3 regions of China—Eastern, Central and Western China [16], between 25^th^ October and 26^th^ December 2019. We used a multistage stratified sampling method. At the first sampling stage, we used the province as the sampling unit, we selected Zhenjiang (Eastern China), Henan (Central China) and Shaanxi (Western China). At the second stage, we sampled one city by province, namely Hangzhou, Zhengzhou and Xi’an. At the third stage, we selected at least 2 districts per city with a final inclusion of 9 districts. At the final stage, we took a convenience sample of individual residents within the community or town. The formula for calculating the sample size for this study is:

$$N=\frac{{\mu }_{\alpha /2}^{2}P\left(1-P\right)}{{\delta }^{2}}$$

$N$ is the sample size; ${\mu }_{\alpha /2}^{2}$ refers to the statistic of 1.96 for a two-sided test with a confidence interval of 95%, $\delta$ is the permissible error and *P* is the rate of willingness to donate organs. Based on a willingness to donate of 18.8% to 47.9% [17, 18], an error ($\delta$) of 3%, α = 0.05, 1 − β = 90%, and an expected 20% nonresponse rate, we estimated the sample size for each site of this study to be between 781 and 1278. In this study, we actually recruited a total of 4305 participants.

### Research instruments

We employed a self-designed questionnaire based on previous researches [19–24] consisting of 4 parts. (1) The participants’ demographic and socioeconomic characteristics, including gender, age, education, marital status, employment, monthly income. (2) Participants’ knowledge of organ donation which includes ten statements with a true/false response option. For items 1 to 7 and 10, the true answer scored 0, whereas the false answer would score 1; for items 8 and 9, the opposite applied. Hence, the sum of the responses represents the total score of knowledge of organ donation and ranges between 0 and 10. (3) Further, we collected information on participants’ attitudes towards organ donation with 20 items using a 5-step Likert scale ranging from fully agree, mostly agree, neutral, mostly disagree, fully disagree. Items 11 to 14 and 30 were scored in declining order where ‘fully agree’ was equal to 4 and ‘fully disagree’ was equal to 0, items 15 to 29 were scored inversely and ‘fully agree’ was scored as 0. The total score of attitudes towards organ donation ranged between 0 and 80. Moreover, the attitudes were grouped in three categories: life view (item 11 to 18), family value (item 19 to 21), and evaluation (item 22 to 30). (4) Lastly, we collected the participants’ willingness to organ donate by asking ‘Are you willing to donate your organs?’ which generated a binary variable (0 = No; 1 = Yes). The specific questionnaire is showed in the appendix (additional file 1). The questionnaire was developed in the following steps: 1) based on previous literature and research, we drafted the first version of the questionnaire, and then organised two rounds of expert consultations, inviting six experts from the subject areas of epidemiology, health statistics and public administration in each round to revise the content of the questionnaire in terms of necessity, feasibility, and logic. 2) A pilot survey was conducted with a sample of 100 residents in Zhengzhou city and Xi^’^an city, respectively; 3) before performing the data analysis, we tested the reliability and validity of questionnaire [25, 26]. The Cronbach α for the questionnaire was 0.740 and internal consistency of instruments was deemed satisfactory. Exploratory factor analysis in structural validity was used to support the validity of the questionnaire. The Kaiser–Meyer–Olkin (KMO) statistic was calculated as 0.862, which passed the Bartlett’s test of sphericity (*χ*^*2*^ = 5556.84, *P* < 0.001), indicating that this data was well suited for factor analysis. Finally, a principal component analysis was carried out to delete and retain entries.

### Quality control

Local investigators were instructed on the study procedures and trained by experts from the Liverpool School of Tropical Medicine, Wenzhou Medical University, Hangzhou Normal University, Xi’an Jiaotong University and Zhengzhou University on how to conduct interviews with study participants. We unified inquiry methods before the formal investigation. Regular assessments and examinations were performed during the entire investigation period.

### Statistical analyses

The questionnaire data were entered into the EpiData 3.1 software (developed by EpiData Association, Odense, Denmark), we used a double entry method for all data. All questionnaire data were checked for outliers prior to data analysis, outliers of all variables used in this study and missing value of outcome variable were dropped. Continuous variables were summarized as means with standard deviations, and categorical variables were summarized as counts and percentages. We compared differences in knowledge, attitudes, and willingness to organ donation by conducting chi-squared test. We assessed the relationship of knowledge and attitudes with the willingness to organ donate using binary logistic regression models. We controlled for the following confounding factors: participants’ gender, age, marital status, education, and monthly income. We present the odds ratios (ORs) with 95% confidence intervals (CIs) and a two-tailed *p*-value of < 0.05 was considered statistically significant. The statistical analyses were performed in SAS 9.4 (SAS Institute, Cary, NC, USA) and figures were made using the R studio software.

## Results

### Basic information of participants

At baseline we recruited 4500 participants from 3 sites into the study of which 4274 finished the questionnaires, resulting in response rate of 94.98%. Table 1 shows the demographic and socioeconomic characteristics of the study participants by region. Participants were recruited in similar numbers from each region, namely 32.59% (1393) from Western China, 32.64% (1395) from Central China and 34.77% (1486) from Eastern China. Similarly, the distribution between urban and rural participants was 47.13% and 52.87%, respectively. A small majority was female (56.68%) as compared to males (43.32%) and mean age of participants was 32.07 ± 12.08 years. The prevalence rate of willingness to donate organs in this study was 47.45% (95%CI: 45.94%, 48.96%).

**Table 1:** Demographic characteristics

Variables	Group	West (*N* = 1393)	Central (*N* = 1395)	East (*N* = 1486)	All (*N* = 4274)
Residence	Urban	498(36.03%)	753(54.06%)	757(50.94%)	2008(47.13%)
	Rural	884(63.97%)	640(45.94%)	729(49.06%)	2253(52.87%)
Gender	Male	545(39.32%)	638(46.16%)	660(44.41%)	1843(43.32%)
	Female	841(60.68%)	744(53.84%)	826(55.59%)	2411(56.68%)
Age (years)	Younger than 31	622(46.77%)	723(52.74%)	884(59.61%)	2229(53.27%)
	31–40	376(28.27%)	364(26.55%)	304(20.50%)	1044(24.95%)
	Older than 40	332(24.96%)	284(20.71%)	295(19.89%)	911(21.77%)
Marital status	Others	407(29.43%)	735(52.80%)	892(60.07%)	2034(47.75%)
	Married	976(70.57%)	657(47.20%)	593(39.93%)	2226(52.25%)
Education	Less than Primary school	88(6.34%)	56(4.02%)	87(5.86%)	231(5.41%)
	Middle-High school	642(46.29%)	485(34.79%)	473(31.85%)	1600(37.51%)
	More than University	657(47.37%)	853(61.19%)	925(62.29%)	2435(57.08%)
Employment	No	909(65.49%)	740(53.12%)	902(60.70%)	2551(59.78%)
	Yes	479(34.51%)	653(46.88%)	584(39.30%)	1716(40.22%)
Monthly income (Ren Min Bi)	Less than 3300	818(60.59%)	613(44.32%)	651(43.81%)	2082(49.35%)
	3300–5999	331(24.52%)	452(32.68%)	390(26.24%)	1173(27.80%)
	6000–9999	137(10.15%)	238(17.21%)	267(17.97%)	642(15.22%)
	More than 9999	64(4.74%)	80(5.78%)	178(11.98%)	322(7.63%)

### Knowledge of organ donation

The knowledge about organ donation mean score (± SD) was 6.50 ± 1.62 out of 10, participants that were willing to organ donate had a higher score as compared to the ones not willing to donate, 6.71 and 6.32, respectively (Table 2). Most participants were aware of the following items 4 and 3: not any doctor can determine brain death (88.53%) and it is correct that living organs can only be donated to immediate family members (80.35%). More than 60% of the participants chose the correct definitions related to organ donation (item 1, 60.90%) and brain death (item 2, 64.87%). A minority of the participants did not agree with the statement that ‘organ removal must be performed only after brain death is determined’ (item 5), indicating a lack of knowledge about the donation procedure. Participants that were willing to donate organs were more likely to know about regulations about the age of an organ donor (item 7) as compared to participants who were not willing to donate (71.49% vs 61.33%, *P* < 0.001). However, for items 1 to 4, 8 and 9 participants that were willing to donate scored lower compared to participants that were not willing to donate in particular for items (from) compared with the participants who were not willing to donate organs (*P* < 0.001).

**Table 2:** The distribution of knowledge on willingness to organ donation

Items	Answer	Willingness to organ donation			*P*-Value
		All (*N* = 4274)	No (*N* = 1393)	Yes (*N* = 1395)	
Item 1: Organ donation refers to donation of cadaveric organs, living organs cannot be donated	True	1669(39.10%)	538(38.68%)	643(46.19%)	< 0.001
	False	2600(60.90%)	853(61.32%)	749(53.81%)	
Item 2: Brain death means that the patient cannot breathe, and the heart cannot beat	True	1501(35.13%)	477(34.24%)	596(42.75%)	< 0.001
	False	2772(64.87%)	916(65.76%)	798(57.25%)	
Item 3: Living organs can only be donated to immediate family members	True	839(19.65%)	269(19.35%)	372(26.70%)	< 0.001
	False	3430(80.35%)	1121(80.65%)	1021(73.30%)	
Item 4: Any doctor can determine brain death	True	490(11.47%)	165(11.84%)	189(13.58%)	0.008
	False	3781(88.53%)	1228(88.16%)	1203(86.42%)	
Item 5: Organ removal must be performed only after brain death is determined	True	2460(57.65%)	855(61.51%)	759(54.57%)	0.008
	False	1807(42.35%)	535(38.49%)	632(45.43%)	
Item 6: People with any disease can donate organs	True	599(14.03%)	199(14.30%)	213(15.31%)	0.103
	False	3670(85.97%)	1193(85.70%)	1178(84.69%)	
Item 7: People of any age can donate organs	True	1406(33.00%)	536(38.67%)	396(28.51%)	< 0.001
	False	2855(67.00%)	850(61.33%)	993(71.49%)	
Item 8: Citizens have not expressed their disapproval of organ donation during their lifetime. After their death, spouses, adult children, and parents can jointly express their consent to organ donation	True	2415(56.56%)	833(59.84%)	752(54.02%)	0.007
	False	1855(43.44%)	559(40.16%)	640(45.98%)	
Item 9: Organ donors cannot claim any monetary compensation	True	2402(56.24%)	868(62.36%)	737(52.91%)	< 0.001
	False	1869(43.76%)	524(37.64%)	656(47.09%)	
Item 10: Donors pay for organ removal surgery	True	801(18.75%)	267(19.18%)	265(19.02%)	0.724
	False	3470(81.25%)	1125(80.82%)	1128(80.98%)	
Total score of knowledge to organ donation	Mean(SD)	6.50(1.62)	6.32(1.66)	6.71(1.53)	< 0.001

### Attitudes to organ donation

The overall mean (± SD) score of attitudes to organ donation in this study was 47.32 ± 9.55, among participants who were willing to donate organs the attitude score was higher as compared to participants not willing to donate (Table 3, *P* < 0.001).

**Table 3:** The distribution of attitude of life view on willingness to organ donation

Variables	Willingness to organ donation [Mean(SD)]			*P*-Value
	All (*N* = 4274)	No (*N* = 1393)	Yes (*N* = 1395)	
The score of attitude of life view on willingness to organ donation (from item 11 to 18)	23.03(6.07)	21.00(5.75)	25.27(5.62)	< 0.001
The score of attitude of family value on willingness to organ donation (from item 19 to 21)	5.78(2.34)	5.44(2.35)	6.14(2.29)	< 0.001
The score of attitude of self-evaluation on willingness to organ donation (from item 22 to 30)	18.46(4.18)	17.84(3.99)	19.15(4.28)	< 0.001
Total score of attitude to organ donation (from item 11 to 30)	47.32(9.55)	44.34(8.99)	50.60(9.05)	< 0.001

**Fig. 1:**
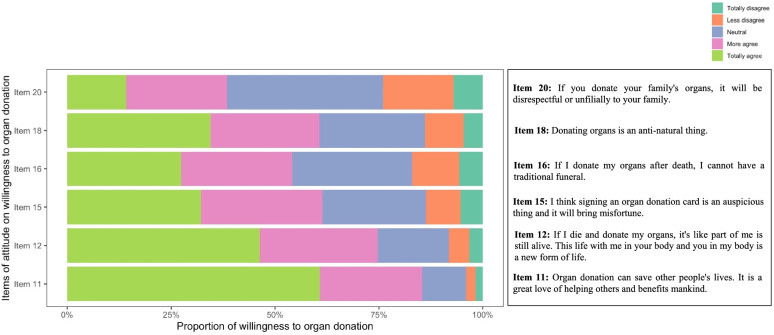
Proportion of willingness to organ donation among different attitudes

Figure 1 showed participants willing to donate organs were more likely to fully agree with the views on organ donation such as ‘organ donation can save lives and benefits mankind’ (item 11; 70.14%) and ‘organ donation is a new form of life’ (item 12; 57.93%). On the other hand, 44.31% of participants that were willing to donate fully disagreed with the statement: ‘I think signing an organ donation card is an auspicious thing and it will bring misfortune’ (item 15), 47.28% disagreed with ‘donating organs is an anti-natural thing’ (item 18) and 38.80% fully disagreed with ‘If I donate my organs after death, I cannot have a traditional funeral’ (item 16). This was 2.5 times higher than the responses by participants who were not willing to donate organs (16.81%). Lastly, 21.43% of participants who were willing to donate organs fully disagreed with ‘If you donate your family's organs, it will be disrespectful or unfilially to your family’ (item 20).

### Association of knowledge and attitudes with willingness to organ donation

**Fig. 2:**
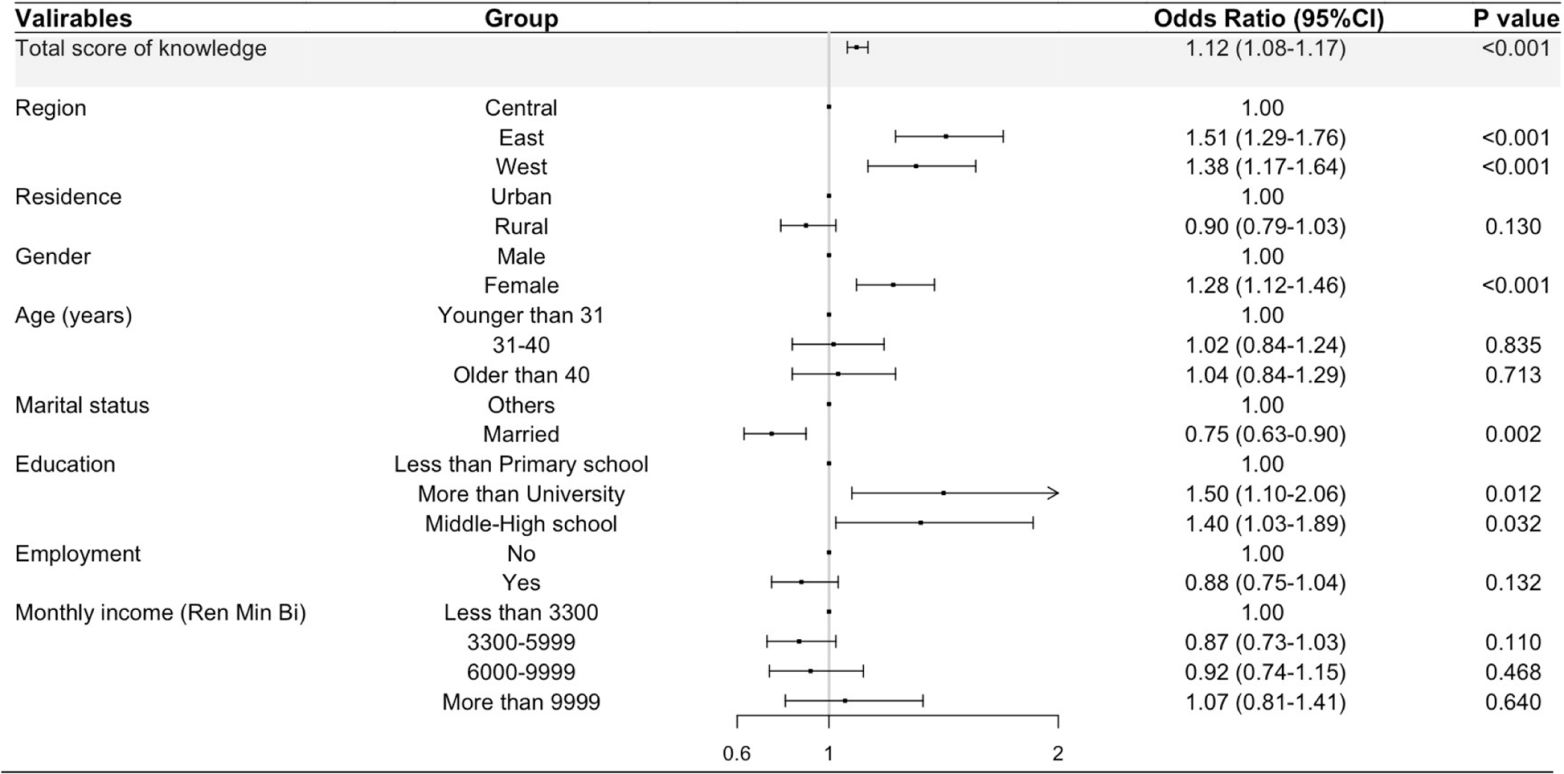
Association of knowledge with willingness to organ donation when controlling for other confounders by multivariate analysis

**Fig. 3:**
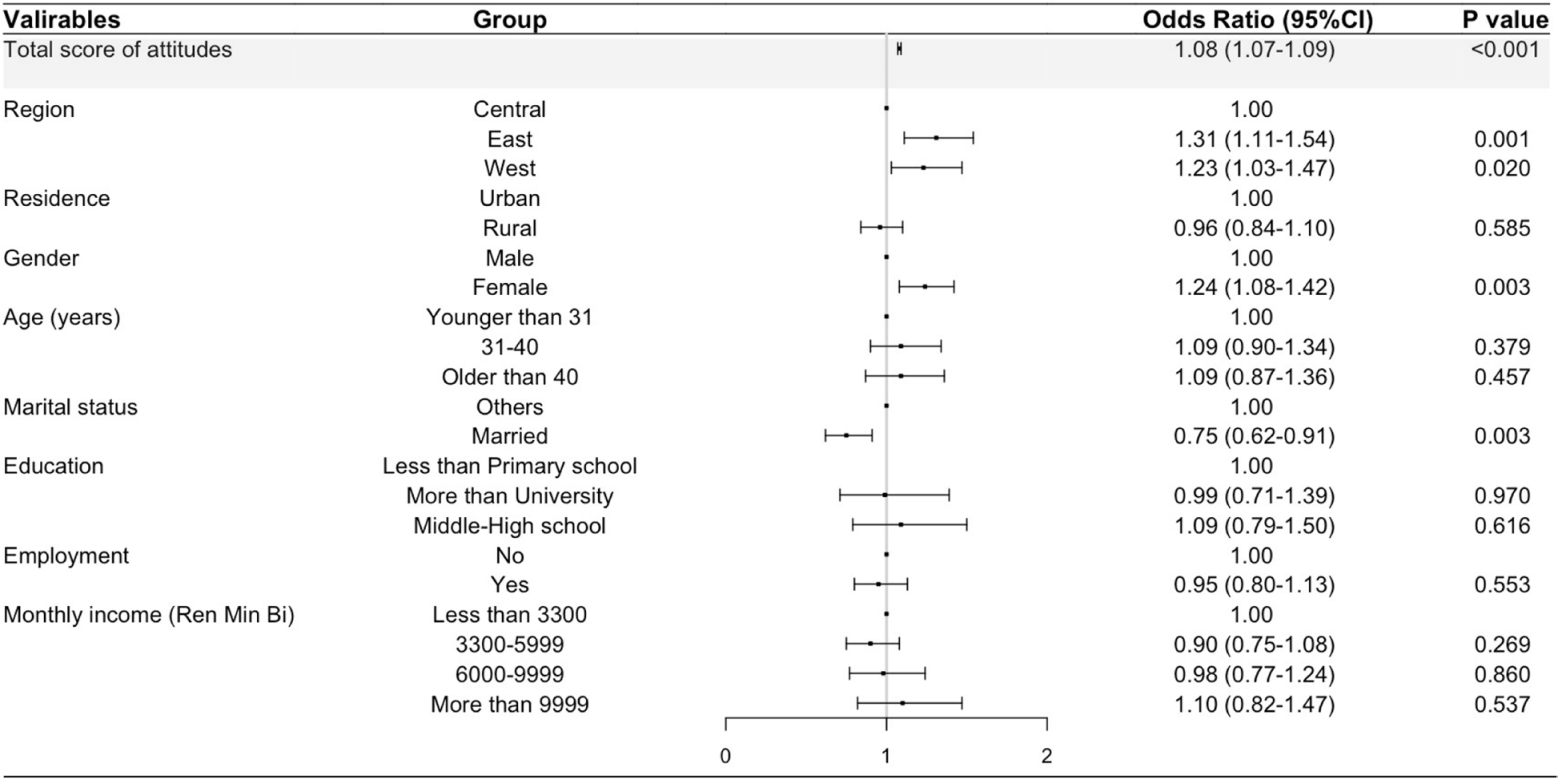
Association of attitudes with willingness to organ donation when controlling for other confounders by multivariate analysis

The association of knowledge about organ donation and the willingness to organ donate is show in Fig. 2. The logistic regression model shows that the sum of knowledge items had the strongest positive association with the willingness to organ donation, among the factors including participants’ residence, gender, age, education, employment, marital and economic status analyzed in this study. The OR of the association of knowledge and willingness was 1.12 (95%CI: 1.08, 1.17; *P* < 0.001), indicating that the knowledge score increases by 1 point, the odds of willingness to organ donation would increase by 12%, meaning more participants will be a potential donor. In addition, the logistic regression model indicated that the attitudes score (OR = 1.08, 95%CI: 1.07, 1.09; *P* < 0.001) was positive determinant of willingness to organ donate (Fig. 3) when controlling for other factors.

### Assessment of the moderating effect

Correlations between variables were tested in the Table 4. Without exception, they were all below 0.61. The moderating effect of knowledge on the attitude–willingness link and gender on the attitude–willingness link was tested by moderated regression analysis (Table 5). At the first step, the total scores of attitude and knowledge were entered as independent predictors of willingness to organ donation when controlling for region, residence, gender, age, marital status, education, employment and monthly income. Table 5 shows that when the total score of attitude towards organ donation is increased by 1 score, the odds of willingness to donates one’s organs would increase by 8.0% (OR = 1.080, *P* < 0.001). In the second step, their interaction term was entered as independent predictors when controlling for other factors. The odds of willingness to organ donation would increase only by 0.4% (OR = 1.004, *P* < 0.001) when the interaction term (total score of knowledge × total score of attitudes) is increased by one unit.

**Table 4:** Correlations between variables

Variable	Willingness to organ donation	Region	Residence	Gender	Age	Marital status	Education	Employment	Monthly income	Total score of knowledge	Total score of attitudes
Willingness to organ donation	1.00										
Region	0.05	1.00									
Residence	-0.02	-0.12	1.00								
Gender	0.08	-0.04	0.04	1.00							
Age	-0.09	-0.09	0.01	-0.11	1.00						
Marital status	-0.11	-0.25	0.04	-0.08	0.61	1.00					
Education	0.09	0.10	-0.19	0.07	-0.47	-0.36	1.00				
Employment	-0.09	0.04	-0.09	-0.25	0.28	0.33	-0.07	1.00			
Monthly income	-0.05	0.17	-0.14	-0.28	0.17	0.26	0.04	0.54	1.00		
Total score of knowledge	0.11	0.05	-0.04	0.07	-0.26	-0.20	0.26	-0.11	-0.04	1.00	
Total score of attitudes	0.32	0.06	-0.06	0.10	-0.19	-0.16	0.24	-0.14	-0.07	0.28	1.00

**Table 5:** Moderated regression analyses to predict whether individual is willing or not willing to donate organs, when controlling for other confounders

Variable	OR	95%CI		*P*
		Lower	Upper	
Step 1				
Total score of knowledge	1.018	0.976	1.063	0.404
Total score of attitudes	1.080	1.070	1.090	< 0.001
Step 2				
Total score of knowledge × Total score of attitudes	1.004	1.003	1.005	< 0.001
Step 3				
Gender (Female)	1.253	1.089	1.440	0.002
Total score of attitudes	1.079	1.070	1.089	< 0.001
Step 4				
Gender (Female) × Total score of attitudes	1.015	1.013	0.0180	< 0.001

Regression analyses in Step 1 and step 2 controlled for region, residence, gender, age, marital status, education, employment, and monthly income. Step 3 and step 4 controlled for total score of knowledge, region, residence, gender, age, marital status, education, employment, and monthly income

In the third step, the total score of attitude and gender were entered as independent predictors of willingness to organ donation when controlling for total score of knowledge, region, residence, gender, age, marital status, education, employment, and monthly income. Compared with male participants, the odds of willingness to partake in organ donation were nearly 1.3 times higher for female participants (OR = 1.253, *P* = 0.002). When the total score of attitude toward organ donation increases by 1 score, the odds of willingness to organ donation would increase by 7.9% (OR = 1.079, *P* < 0.001). In the fourth step, their interaction term was entered as independent predictors. Compared with male participants, the odds of willingness to partake in organ donation would increase by 1.5% for female when the total score of attitudes is increased by 1 score (OR = 1.015, *P* < 0.001). Moderated regression analyses revealed that there was an interaction effect on willingness to organ donation between knowledge and attitude, gender and attitude.

## Discussion

The number of organs donated in China has risen rapidly over the past decade but the need is not met which presents a major obstacle to saving lives. This study is the latest survey of the general public’s knowledge, attitudes, and willingness to organ donate across geographical settings in Western, Central and Eastern China. We provide an important perspective on organ donation and the barriers to willingness to donate related to knowledge and attitudes.

### Main findings

In this study, the general public’s rate of willingness to organ donation was 47.45%. For the domestic public, this rate was similar to the rate in Nanning City (47.92%) [18], higher than the rate in Ji’nan city (46%) [27], Zhejiang Province (18.8%) [17] and Northwest China (29.5%) [28]; Compared with foreign data, this rate was higher than the rate in Japan (41.9%) [29], and lower than the rates in Syria (62%) [30] and in the Middle East (49.8) [31].

Unsurprisingly, the rate of the general public’s willingness to donate organs was lower than that previously reported in Chinese health professionals (49.3%) [14], Chinese transplantation patients and their caregivers (62.7%) [32], medical students in Spain (79%) [33], in Germany (63.5%) [34] and Jimma University (58.1%) [35]. All things considered, general public’s willingness to donate organs in this study is not particularly high and targeted measures should be implemented by the policy-makers and scientists to improve the situation.

This study shows that higher knowledge about organ donation was associated with the willingness to become an organ donor, which is consistent with previous studies conducted by Figueroa CA et al. in Dutch and Wale J et al. in the United Kingdom [36, 37]. Other studies conducted in Australia, Korea, Niger and Ghana showed no such association [38–41]. These discrepancies might be related not only to the measurement of knowledge (the content and number of questions), but also to different cultural and country specific factors such as traditional values, religious beliefs, compensation mechanisms, institutional credibility and ideals [42]. The relationship between knowledge and willingness to organ donation needs more research to verify. In this study, 10 questions were designed to determine general public’s basic knowledge about organ donation. Only 60.90% of the general public identified the right meaning of brain death, and 42.35% of general public were familiar with the right procedure of organ removal. Therefore, increasing knowledge about the concept of brain death and the transplant procedure may help raise the rate of willingness to organ donation in China. Our study also indicated that attitudes were positively associated with the willingness to organ donation, which was confirmed with other studies [21]. Raising public's awareness of organ donation and changing their attitudes towards it will hopefully increase their willingness to donate, and that is where narrative medicine fits in. It has been justified in practice that narrative medicine enables doctors to communicate with patients more effectively, and is especially suitable for difficult doctor-patient communication, esp. in organ donation communication to reduce family members’ lack of understanding to organ donation [43]. It is important to note, that most of general public were strongly in favour of organ donation. Chinese traditional values such as donating organs was an auspicious thing (item 15), an anti-natural thing (item 18), cannot have a traditional funeral (item 16), was disrespectful to family (item 20) had not hindered Chinese general public’s intentions about organ donation. This finding was consistent with Zhang H, et al.’s and Aijing L, et al.’s investigations after 2015, but contrary to several other studies in China before 2014 [42, 44, 45]. A possible explanation is that with the development of science and education, Chinese general public’s attitudes to death and organ donation have changed. From the dimension of evaluation, 63.4% of general public support ‘organ donor families can receive appropriate financial assistance for organ donors’ families, which was consistent with Rasiah R, et al. and Gordon EJ, et al.’s conclusion that financial incentives were significant to help raise rate of willingness to organ donation [46, 47].

The present data provide some evidence for the moderating role of knowledge and gender in predicting willingness to organ donation. More specifically, the association between attitudes towards organ donation and willingness to donate organs appeared to be stronger for participants with high total knowledge scores compared to those with low total knowledge scores. Effective measures to increase the willingness to donate organs should not only improve the public’s attitude towards organ donation, but also increase their knowledge about organ donation. Additionally, the link between attitudes toward organ donation and willingness to organ donation appeared to be stronger for women compared to men.

The influence of gender may be related to gender stereotypes. According to this notion, women should feel a strong moral obligation to become a potential organ donor and they have a stronger sense of compassion than men [48]. The results of our study also suggested a lower rate of willingness to donate organs among those who were married, which was consistent with Abukhaizaran and Yan’s studies [49, 50], but in contrast to Iliyasu’s study [51]. This association remains to be explored by further research.

### Limitations

This study has some limitations. Firstly, this is an observational study and the confounders of willingness to organ donation included in this study are limited by the pre-specified questions in the surveys. There could be some potential unobserved confounding factors (related policies such as presumed consent law and allocation priority were found to be effective measures to increase organ donation [52–54]) we did not control for in the logistic model. Secondly, this study reports the influence of general public’s knowledge and attitudes on willingness to organ donation based on a quantitative study, more evidence based on qualitative studies and randomized controlled trials are needed to support the results comprehensively. Nevertheless, this study forms an important baseline step for future studies.

## Conclusions

In summary, knowledge and attitudes were found to be positively associated with Chinese general public’s willingness to organ donation and their attitudes were less hindered by Chinese traditional values. Besides, our study suggested that a donation education program focusing on increasing knowledge about the concept of brain death and the transplant procedure may help raise the rate of willingness to organ donation in China, and ultimately to reduce the imbalance between the supply and need for organ transplantation.

## Supplementary Information

**Additional file 1. **

## Data Availability

The datasets generated for the current study are available from the corresponding author on reasonable request.

## Abbreviations

CI: Confidence Interval
ICU: Intensive Care Unit
OR: Odds Ratio
WHO: World Health Organisation
SD: Standard Deviation
