# Supplementary material for: Knowledge, attitudes and willingness to organ donation among the general public: a cross-sectional survey in China
Source: BMC Public Health. 2022 May 9;22:918. doi: 10.1186/s12889-022-13173-1 (PMC9082919; doi:10.1186/s12889-022-13173-1)
Supplement: Supplementary file 1 — Additional file 1. [file 12889_2022_13173_MOESM1_ESM.docx]

**Questionnaire for the general public’ knowledge, attitude and willingness**

**to human organ donation in China**

Family address：_______________ City _____________District _______________Street _____________ Community_____________（detailed address）

Name：__________________ Phone number：__________________

City administrative division code□ District administrative division code □ Street code □ Community code□□ Personal code□□ Total code□□□□□

The start date of the survey：2019 (month) (day)

The end of the survey ：2019 (month) (day) Investigator（signature） ：_____ _________

Date of verification ：2019 (month) (day) Instructor of investigation（signature）：______________

**Informed consent**

Dear interviewee：

We sincerely invite you to participate in research on the construction of ethics system for human organ donation. The purpose of this study is to understand the knowledge, attitude and practice of Chinese residents on human organ donation, which improves the rate of organ donation, and save millions of patients with organ failure. Since your condition meets the inclusion criteria for this study, we invite you to join this study. Your participation in this research is voluntary. This study has been reviewed by the Institutional Ethics Review Committee. If you agree to participate in this study, please see the instructions below.

**Research Process and Method**：

Please answer the question one by one. There are no wrong answers, you just need to answer the question in detail and truthfully. Any questions in the process of answering can be asked to the researcher. If you have any discomfort during the process of answering, please timely feedback to us. After completing the answer, please submit the questionnaire in time. Please confirm after the researcher is qualified.

**Possible benefits of research**：

At present, the low rate of organ donation is the main problem restricting organ transplantation in China. This study is intended to increase the rate of organ donation, so it is important to do this research well. Respondents may obtain organs and save lives if they need them.

**Research risks and discomforts**：

This research will not cause any harm to your body. The questionnaire will involve some sensitive issues, such as your age, gender, etc. We will keep this information strictly confidential, so you can rest assured to fill out the questionnaire.

**Privacy issues：**

If you decide to participate in this study, your participation and the personal data in the survey are confidential. For example: Your identity and personal information will not be disclosed to members outside the research team unless your permission is obtained. To ensure that research is carried out in accordance with regulations, members of government administrations or ethics review committees can access your personal data at the research unit as required. When the results of this study were published, a commitment to confidentiality was also required. As a subject, you can keep informed of the information and research progress related to this study, and voluntarily decide whether to participate or not. You can choose to notify the investigator to withdraw from the study at any time, and your data will not be included in the study results.

**Contact information：**

If you have questions related to this research, or if you have any discomfort or injury during the research, or if you have questions about the rights of participants in this research, you can contact the research leader at 0577-86689920.

**Informed consent signature：**

By submitting this form, you are indicating that you have read the description of the study, are over the age of 16, and that you agree to the terms as described.

Interviewee signature：_____________________ Date：_____year ______month _______day

Investigator signature：_____________________ Date：_____year ______month _______day

（*Note: if the subject is illiterate, a witness's signature is required, if the person is incapacitated, the agent's consent is required）*

**Table 1. Demographical and Socioeconomic Questions**

| No. | Questions and options | Answer |
| --- | --- | --- |
|  | The category of respondents? ① General public ② Donor's family ③ Organ donation coordinator ④ Medical staff ⑤ Member of the Medical Ethics Review Committee |  |
|  | Resident type：① Urban ② Rural |  |
|  | Gender：① Male ② Female |  |
|  | Date of birth ：______year _____month _____ day |  |
|  | Ethnicity：① Chinese Han ② Minority |  |
|  | Marital status：① Single （Jump to question 8）② Married ③ Divorced ④Widowed ⑤ Cohabitation |  |
|  | Number of children：① 0 ② 1 ③ 2 ④ 3 ⑤ ≥4 |  |
|  | Are you the only child in your family？ ① Yes ② No |  |
|  | Educational background：① less than primary school （Jump to question 11） ② Middle and high school（including secondary education, Jump to question 11） ③ University and above |  |
|  | Major：① Medical ② Other |  |
|  | Religion：① None ② Buddhism ③ Taoism ④ Christianity ⑤ Islam ⑥ Catholic ⑦ Others (Please specify_________) |  |
|  | Occupation：① Student ② Housekeeper ③ Employees of enterprises and institutions/ Civil servant ④ Self-employed persons ⑤ Rural migrant workers ⑥ Retirement ⑦ Other (Please specify_________) |  |
|  | Which of the following is your monthly income range? (Chinese yuan) （According to the per capita monthly income of Chinese urban residents in 2018 of 3,271 yuan）① < 3300 ② 3300 -5999 ③ 6000 -9999 ④ >10000 |  |

**Table 2. Personal experience questions**

| No. | Questions and options | Answer |
| --- | --- | --- |
|  | Do you understand of organ donation? ① Yes, very well ② Yes, some ③ a little bit ④ Never heard (If you choose ④, then jump to question 25) |  |
|  | If so, where did you get your knowledge? ①Newspaper/book ②TV/radio ③The Internet ④Medical institution ⑤Other ways_____________ |  |
|  | Which medical related media do you mainly focus on? ①Television program ②Network program ③WeChat public account/Weibo account ④Newspapers, magazines and brochures |  |
|  | How do you feel about the publicity of organ donation in the selected media? ①Good ②Average ③Bad ④I do not know |  |
|  | How often do you see information/news about organ donation on average? ①A year or more ②Six months to a year ③One month to six months ④No more than a month |  |
|  | Would you be interested in organ donation due to media coverage? ①No ②Maybe ③Yes (If you choose ①, then jumpp to question 24) |  |
|  | What information/news are you most impressed with about organ donation? ①Reports of individual organ donation ②Current status of organ donation ③Ways and procedures for organ donation ④Others_____________ |  |
|  | Whether you have participated in the dissemination of information about organ donation (such as comments, reposting related Weibo, WeChat articles, etc.)? ① Never participated ② I reposted ③ I commented ④ I reposted and commented |  |
|  | Which media do you think can play a greater role in promoting organ donation? ① Newspapers/magazines/brochures ②Radio/television ③ Internet (such as portal, microblog, WeChat, etc.) ④Interpersonal communication |  |
|  | What do you think is the most inadequate aspect of the media in raising public awareness of organ donation? ①seriously insufficient, seldom see related information ②News content is monotonous and lacking in new ideas ③Others_____________ |  |
|  | Have you or your relatives or friends had any experience in organ donation：① Yes ② No |  |
|  | Have you ever cared for organ transplant patients? ① Yes ② No |  |
|  | Are there any medical staff in your family? ① Yes ② No |  |
|  | Have you participated in any volunteer activities (not just about organ donation)? ① Yes ② No |  |

**Table 3. KAP questionnaire**

| **A. Knowledge towards organ donation** | | |  | |  | |  | | |  | |  |  |
| --- | --- | --- | --- | --- | --- | --- | --- | --- | --- | --- | --- | --- | --- |
| Please mark "√" at the option you agree with according to the actual situation. "1" indicates approval and "0" indicates opposition. | | | | | | | | | | | | |  |
| Question number and question | | | | True | | | | | False | | | |  |
|  | Organ donation refers to donation of cadaveric organs, living organs cannot be donated | | | True | | | | | False | | | |  |
|  | Brain death means that the patient cannot breathe, and the heart cannot beat | | | True | | | | | False | | | |  |
|  | Living organs can only be donated to immediate family members | | | True | | | | | False | | | |  |
|  | Any doctor can determine brain death | | | True | | | | | False | | | |  |
|  | Organ removal must be performed only after brain death is determined | | | True | | | | | False | | | |  |
|  | People with any disease can donate organs | | | True | | | | | False | | | |  |
|  | People of any age can donate organs | | | True | | | | | False | | | |  |
|  | Citizens have not expressed their disapproval of organ donation during their lifetime. After their death, spouses, adult children, and parents can jointly express their consent to organ donation | | | True | | | | | False | | | |  |
|  | Organ donors cannot claim any monetary compensation | | | True | | | | | False | | | |  |
|  | Donors pay for organ removal surgery | | | True | | | | | False | | | |  |
| **B．Attitude towards Organ donation** | | | | | | | | | | | | |  |
| Please tick "√" at the option that best fits the situation according to the real situation.  "1" means strongly agree, "2" means slightly agree, "3" means neutral, "4" means slightly disagree, "5" means strongly disagree | | | | | | | | | | | | |  |
| Question number and question | | totally agree | | more agree | | neutral | | less disagree | | | totally disagree | | |
| **Attitude of life view** | |  | |  | |  | |  | | |  | | |
|  | Organ donation can save other people's lives. It is a great love of helping others and benefits mankind | totally agree | | more agree | | neutral | | less disagree | | | totally disagree | | |
|  | If I die and donate my organs, it's like part of me is still alive. This life with me in your body and you in my body is a new form of life | totally agree | | more agree | | neutral | | less disagree | | | totally disagree | | |
|  | I believe that the human body is only the temporary home of the soul and mind, and can be donated after death | totally agree | | more agree | | neutral | | less disagree | | | totally disagree | | |
|  | Organ donation does not violate my beliefs | totally agree | | more agree | | neutral | | less disagree | | | totally disagree | | |
|  | I think signing an organ donation card is an auspicious thing and it will bring misfortune | totally agree | | more agree | | neutral | | less disagree | | | totally disagree | | |
|  | If I donate my organs after death, I cannot have a traditional funeral | totally agree | | more agree | | neutral | | less disagree | | | totally disagree | | |
|  | If I have to face death, I must die at home, so I will not donate organs | totally agree | | more agree | | neutral | | less disagree | | | totally disagree | | |
|  | Donating organs is an anti-natural thing | totally agree | | more agree | | neutral | | less disagree | | | totally disagree | | |
| **Attitude of family value** | |  | |  | |  | |  | | |  | | |
|  | My family will not be happy if they know I will donate organs after I die | totally agree | | more agree | | neutral | | less disagree | | | totally disagree | | |
|  | If you donate your family's organs, it will be disrespectful or unfilial to your family | totally agree | | more agree | | neutral | | less disagree | | | totally disagree | | |
|  | It is a sign of filial piety to donate organs according to the wishes of the elders | totally agree | | more agree | | neutral | | less disagree | | | totally disagree | | |
| **Attitude of evaluation** | |  | |  | |  | |  | | |  | | |
|  | I'm not sure if the organ I donated will be transplanted to someone in need | totally agree | | more agree | | neutral | | less disagree | | | totally disagree | | |
|  | Although organ donation can save lives, it does not always work well | totally agree | | more agree | | neutral | | less disagree | | | totally disagree | | |
|  | I don't think that my age or current physical condition is suitable for organ donation after death | totally agree | | more agree | | neutral | | less disagree | | | totally disagree | | |
|  | If I agree to donate my organs, I don't think my body will be respected | totally agree | | more agree | | neutral | | less disagree | | | totally disagree | | |
|  | If I say yes, I'm afraid the doctors will rush to remove my organs before my death is assured | totally agree | | more agree | | neutral | | less disagree | | | totally disagree | | |
|  | I'm afraid I will be found to have some diseases due to organ donation | totally agree | | more agree | | neutral | | less disagree | | | totally disagree | | |
|  | If you or a family member donates an organ, want to know who to donate and keep in touch with | totally agree | | more agree | | neutral | | less disagree | | | totally disagree | | |
|  | There is a sense of kinship if you know who the organ has been donated to and keep in touch with them | totally agree | | more agree | | neutral | | less disagree | | | totally disagree | | |
|  | Organ donor families can receive appropriate assistance in times of financial difficulty | totally agree | | more agree | | neutral | | less disagree | | | totally disagree | | |
| **C. Organ Donation Willingness** | | | | | | | | | | | | |  |
| Questions and options | | | | | | | | | | | | Answer |  |
|  | Would you like to donate your organs？① No ② Yes | | | | | | | | | | |  |  |
